# Maternal exposure to CeO_2_NPs derails placental development through trophoblast dysfunction mediated by excessive autophagy activation

**DOI:** 10.1186/s12951-022-01334-8

**Published:** 2022-03-15

**Authors:** Zhuxiu Chen, Yanqing Geng, Rufei Gao, Hangtian Zhong, Jun Chen, Xinyi Mu, Xuemei Chen, Yan Zhang, Fangfang Li, Junlin He

**Affiliations:** 1 School of Public Health and Management, Chongqing Medical University, Chongqing, 400016 China; 2 Joint International Research Laboratory of Reproduction & Development, Chongqing Medical University, Chongqing, 400016 China; 3 College of Pharmacy, Chongqing Medical University, Chongqing, 400016 China

**Keywords:** CeO_2_NPs, Trophoblast, Placenta, Autophagy, HTR-8/SVneo

## Abstract

**Background:**

The increasing use of cerium dioxide nanoparticles (CeO_2_NPs) in biomedical field has attracted substantial attention about their potential risks to human health. Recent studies have shown that nanoparticles can induce placental dysfunction and even fetal abortion, but a more detailed mechanism of nanoparticles affecting placental development remains elusive.

**Results:**

Here, we constructed a mouse exposure model with different doses of CeO_2_NPs (2.5, 4, 5, 7.5, and 10 mg kg^−1^ day^−1^, average particle size 3–5 nm), finding that intravenous exposure to pregnant mice with CeO_2_NPs could cause abnormal placental development. Deposited nanoparticles were able to be observed in the placental trophoblast at doses of 5 and 7.5 mg kg^−1^ day^−1^. Diving into molecular mechanisms indicated that CeO_2_NPs exposure could lead to autophagy activation in placental trophoblast. At the cellular level, exposure to CeO_2_NPs inhibited the migration and invasion of HTR-8/SVneo and activated the autophagy through mammalian target of rapamycin complex1 (mTORC1) signaling pathway. Furthermore, inhibition of autophagy initiation by 3-Methyladenine (3-MA) partially restored the function of HTR-8/SVneo, while blocking autophagic flow by Chloroquine (CQ) aggravated the functional damage.

**Conclusions:**

Maternal exposure to CeO_2_NPs impairs placental development through trophoblast dysfunction mediated by excessive autophagy activation. These results suggested that autophagy dysfunction may be a potential mechanism for the impairment of trophoblast by CeO_2_NPs exposure. As above, our findings provide insights into the toxicity mechanism to the reproductive system induced by rare-earth nanoparticles exposure.

**Graphical Abstract:**

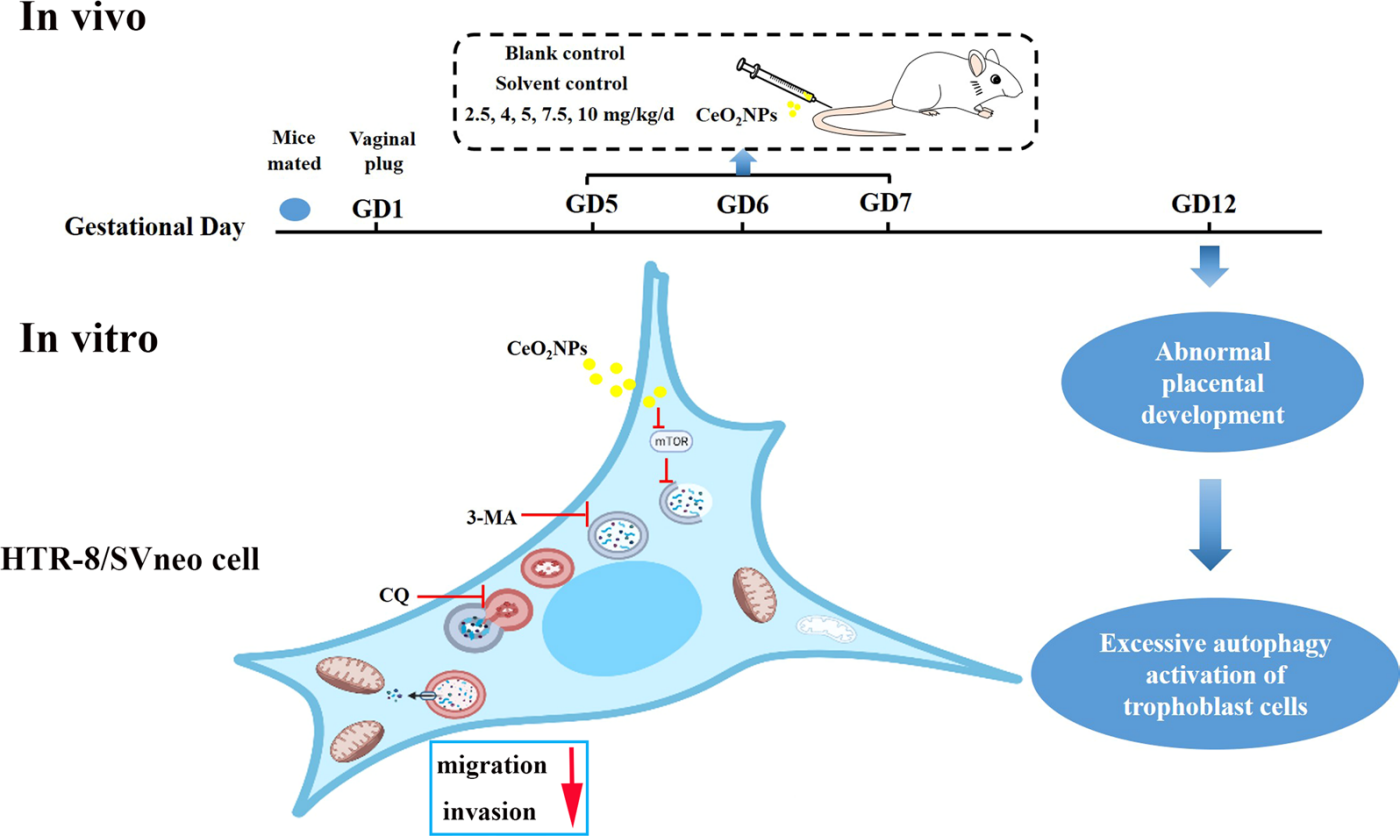

**Supplementary Information:**

The online version contains supplementary material available at 10.1186/s12951-022-01334-8.

## Introduction

Cerium dioxide nanoparticles (CeO_2_NPs) are a kind of important rare-earth nanoparticle with nanomaterials and rare earth properties, which are widely used in commercial and industrial products in recent years, including cosmetics, glass polishing, fuel additives, catalysts and effective carriers of corrosion inhibitors [1, 2]. Benefiting from the high reducibility and low toxicity s of CeO_2_NPs, they have shown promising applications in biology and medicine [3, 4], especially in the treatment of hepatology [5, 6], encephalopathy [7], antithrombic and burn [8, 9]. Moreover, the beneficial effects of CeO_2_NPs treatment are also reflected in neurology, cardiology, ophthalmology and oncology [10–13]. However, studies have reported that exposure of CeO_2_NPs through different pathways seems to result in potential toxicological effects, such as pulmonary inflammation [14, 15], impairment of microvascular smooth muscle signaling [16], and potential genetic damage [17]. Among them, intravenous injection is one of the routes more frequently used in nano-therapy [18]. In reproductive field, nanomedicine delivery systems may provide alternative targeted intervention strategies, treating the source of the disease and minimizing long-term consequences for the mother and/or her fetus [19]. Therefore, it is important to investigate the effect of CeO_2_NPs treatments by intravenous administration. The limited researches on reproductive effect of CeO_2_NPs also restrict its further development in biomedical application. Thus, the biosafety of CeO_2_NPs exposure during pregnancy needs to be further studied.

Several studies have confirmed that nanoparticles such as fine particulate matter (PM_2.5_) [20], Silica and titanium dioxide [21] can cause pregnancy complications, such as poor fetal growth. Normal growth of the embryo during gestation depends on normal placental development [22]. The trophoblast cell lineage is derived from the trophectoderm of the blastocyst, and its differentiation is essential for placental development [23]. Generation of these distinct trophoblast cell types within the placenta is necessary to accomplish the complex physiological processes of maternal–fetal exchange [24]. Of note, abnormal migration and invasion of trophoblast cells may lead to placental development disorders [25]. Furthermore, both inadequate and excessive autophagy have been reported to induce placental dysplasia [26, 27]. Autophagy, a conserved and protective cellular program which degrades unwanted proteins, damaged organelles and foreign matter through the lysosomal degradation pathway to maintain cellular homeostasis in eukaryotic cells [28], has been reported to affect EVT function, trophoblast infiltration, vascular remodeling during normal placental development and plays multiple important roles in embryonic development [29]. Although autophagy dysfunction caused by excessive autophagy activation and inhibition of autophagy flow has been considered as a possible mechanism of nanoparticles-induced toxicity [30–32], the specific roles of autophagy in CeO_2_NPs-induced cytotoxicity, particularly in the trophoblast cell, have not been clarified. Naturally, the effects of CeO_2_NPs exposure on trophoblast cells and its mechanism deserve a thorough study.

In this study, a mouse exposure model with different doses of CeO_2_NPs during pregnancy was constructed to screen the reference range without negative biological effect, which should be less than 4 mg kg^−1^ day^−1^. Our results indicate that exposure to 5 and 7.5 mg kg^−1^ day^−1^ CeO_2_NPs impairs placental development through autophagy dysfunction. Furthermore, we show that exposure to CeO_2_NPs inhibits the migration and invasion of HTR-8/SVneo (human chorionic trophoblast cell line) and provokes autophagy excessively through the mTORC1 signaling pathway. These data provide essential information for the safer use of nanoparticles and allow to better understand the toxic effects of nanoparticles exposure to the reproductive system.

## Results

### Effects of CeO_2_NPs exposure on pregnancy status in mice

**Fig. 1:**
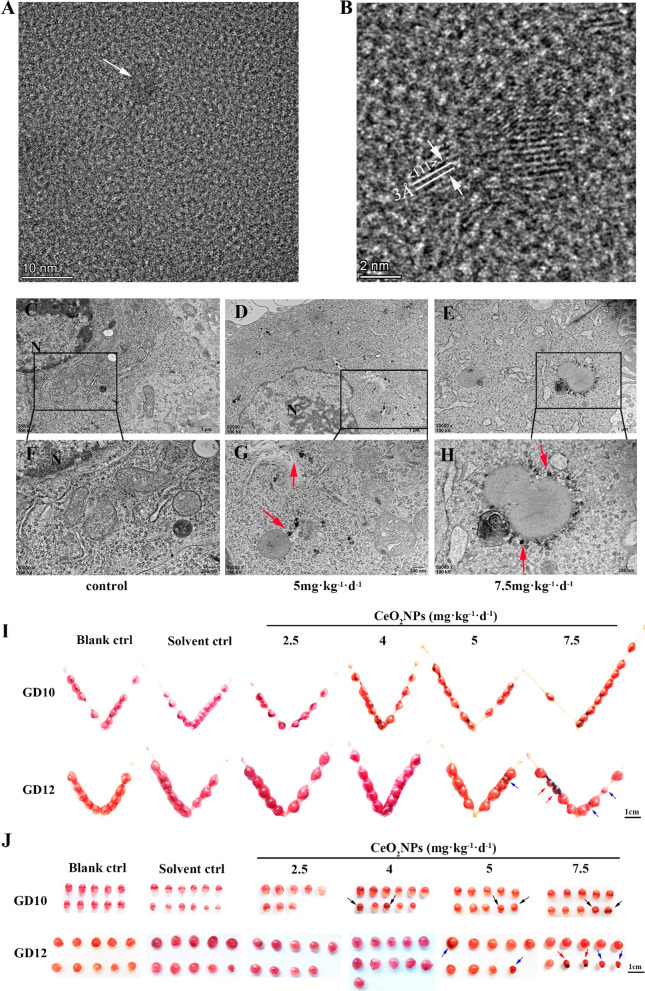
Effects of CeO_2_NPs exposure on pregnancy status in mice. The morphology and size of CeO_2_NPs. **A** High-resolution FE-TEM images of CeO_2_NPs (Scale bar = 10 nm). **B** Lattice fringes characteristics of CeO_2_NPs (Scale bar = 2 nm). Deposition of CeO_2_NPs in placental tissue on GD12 observed by TEM. TEM images of placental tissue of control group (**C**, **F**), 5 mg kg^−1^ day^−1^ CeO_2_NPs-exposed group (**D**, **G**), 7.5 mg kg^−1^ day^−1^ CeO_2_NPs-exposed group (**E**, **H**) on GD12 (Upper scale bar = 1 μm, lower scale bar = 200 nm). N: nucleus, red arrows show cerium dioxide nanoparticles. **I** The uterine appearance on GD10 and GD12 (n = 7, Scale bar = 1 cm). Red arrow indicates embryo absorption with severe uterine bleeding and blue arrow indicates abnormal placenta development on GD12. **J** Representative images of embryo and placentas respectively collected on GD10 and GD12 (n = 7, Scale bar = 1 cm). Black arrow indicates abnormal bleeding of embryo on GD10, red and blue arrow respectively indicate embryo with hemorrhage and stunted placenta

The morphology and size of CeO_2_NPs were observed by Field emission transmission electron microscopy (FE-TEM). Figure 1A showed the higher resolution images of CeO_2_NPs that presented in lattice form (3–5 nm). The lattice fringes with d spacing of 3 Å and the distance between the (111) lattice planes of CeO_2_ are corresponding with the previously report (Fig. 1B) [33]. Our TEM (transmission electron microscopy) analysis confirmed the deposition of CeO_2_NPs in the placenta tissue of exposed gestational mice. CeO_2_NPs were identified in the placental trophoblast cells of both 5 and 7.5 mg kg^−1^ day^−1^ CeO_2_NPs exposed groups, while no particle-like structures were observed in the dose groups below 5 mg kg^−1^ day^−1^ (Fig. 1C–H). The effects of different doses of CeO_2_NPs on the pregnancy process were assessed in detail. First, animal model of pregnant mice was constructed by tail vein injection with different doses of CeO_2_NPs (2.5, 4, 5,7.5 and 10 mg kg^−1^ day^−1^). Pregnant mice died after intravenous injection of CeO_2_NPs (10 mg kg^−1^) once a day on GD5 (gestational day 5) and GD6, indicating that 10 mg kg^−1^ day^−1^ CeO_2_NPs exposure was lethal. Then, pregnancy status of mice were observed. We calculated the uterine and maternal body weight on GD8, GD9, GD10, and GD12, on which are the key periods of placental development in first trimester mice. The results showed that the uterine weight and uterine organ coefficient of mice exposed to CeO_2_NPs had no significant difference compared with that in control group (Additional file 1: Figure S1A–C). There was no significant difference on uterine appearance among the groups on GD8 and GD9 (Additional file 1: Figure S1D). However, local bleeding on the embryo surface was found on GD10 response to CeO_2_NPs exposure (even 4 mg kg^−1^ day^−1^) (Fig. 1I). Embryo reabsorption with uterine bleeding and abnormal placenta development were observed in CeO_2_NPs treatment groups on GD12 (Fig. 1J). These results confirmed that a certain dose of CeO_2_NPs exposure would result in an adverse effect on pregnancy.

### Effects of CeO_2_NPs exposure on placental development

**Fig. 2:**
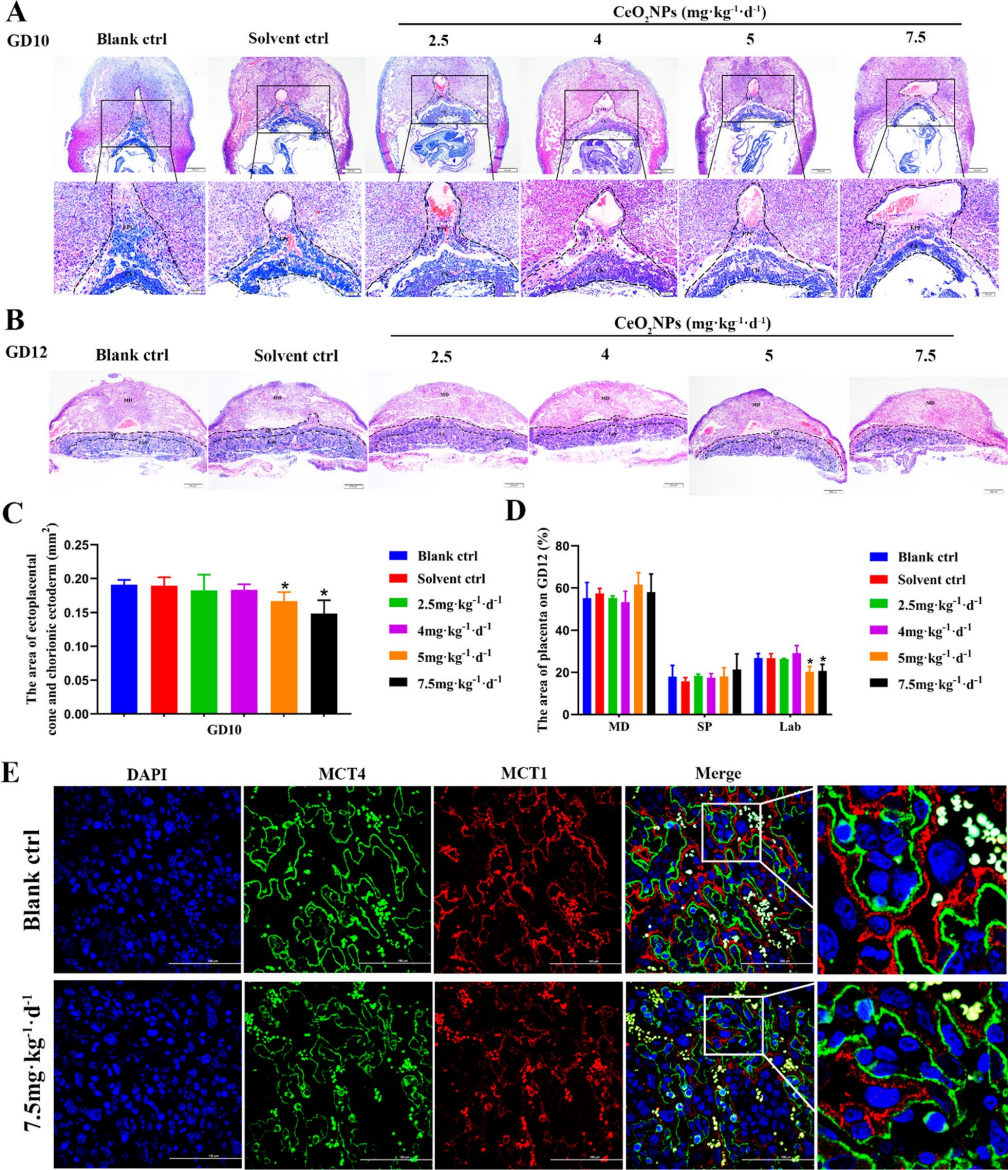
Effects of CeO_2_NPs exposure on early placental development. **A** HE staining of uterine tissue on GD10 observed under light microscope (Upper scale bar = 200 μm, lower scale bar = 50 µm). EPC: ectoplacental cone, Ch: chorionic ectoderm. **B** HE staining of placenta tissue on GD12. MD: maternal decidual, SP: spongiotrophoblast, Lab: labyrinth (Scale bar = 200 μm). **C** The area of ectoplacental cone and chorionic ectoderm on GD10. **D** The area of maternal decidua, spongiotrophoblast and labyrinth of placenta on GD12. *P < 0.05 vs. control. **E** MCT1 and MCT4 immunofluorescence staining of placenta on GD12 (Scale bar = 100 µm)

To clarify the relationship between CeO_2_NPs exposure and placental development, hematoxylin and eosin (H&E) staining were used to examine the pathological histology of the placenta development on GD8 (establishment of decidua), GD9 (endometrial spiral artery remodeling), GD10 (initial of placental circulation), and GD12 (formation of placental structure) in CeO_2_NPs-treated mice. The results showed that no significant changes were observed in decidual tissue morphology of the CeO_2_NPs treated mice on GD8 (Additional file 1: Figure S2A), while treatment with 5 and 7.5 mg kg^−1^ day^−1^ CeO_2_NPs resulted in the decrease of ectoplacental cone and extraembryonic ectoderm area on GD9 (Additional file 1: Figure S2B, C). The total area of ectoplacental cone (EPC) and chorionic ectoderm (Ch) on GD10, which reflect the degree of trophoblast invasion, was reduced after CeO_2_NPs exposure (Fig. 2A, C). The labyrinth area on GD12 decreased significantly in CeO_2_NPs treatment groups, especially in 5 and 7.5 mg kg^−1^ day^−1^ CeO_2_NPs-treated groups (Fig. 2B, D). MCT1 and MCT4, two monocarboxylate transporters that specifically express in the SynT-1 (syncytiotrophoblast-1) and SynT-2 layers [34], respectively, were stained by immunofluorescence to separate fetal blood vessel and maternal blood sinuses. MCT1^+^ SynT-1 and MCT4^+^ SynT-2 are distributed uniformly in the labyrinth layer of placenta in blank control group (Fig. 2E). Notably, the labyrinth layer MCT1^+^ SynT-1 was reduced significantly in 7.5 mg kg^−1^ day^−1^ CeO_2_NPs-treated group, suggesting abnormal maternal vascular remodeling (Fig. 2E). These results indicated that exposure to CeO_2_NPs had an adverse effect on placental development. There were no significant changes in uterus appearance of pregnant mice exposed to lower than 4 mg kg^−1^ day^−1^ CeO_2_NPs, speculating that the safe dose of CeO_2_NPs should below 4 mg kg^−1^ day^−1^.

### CeO_2_NPs exposure activated autophagy of placental trophoblast in mice

**Fig. 3:**
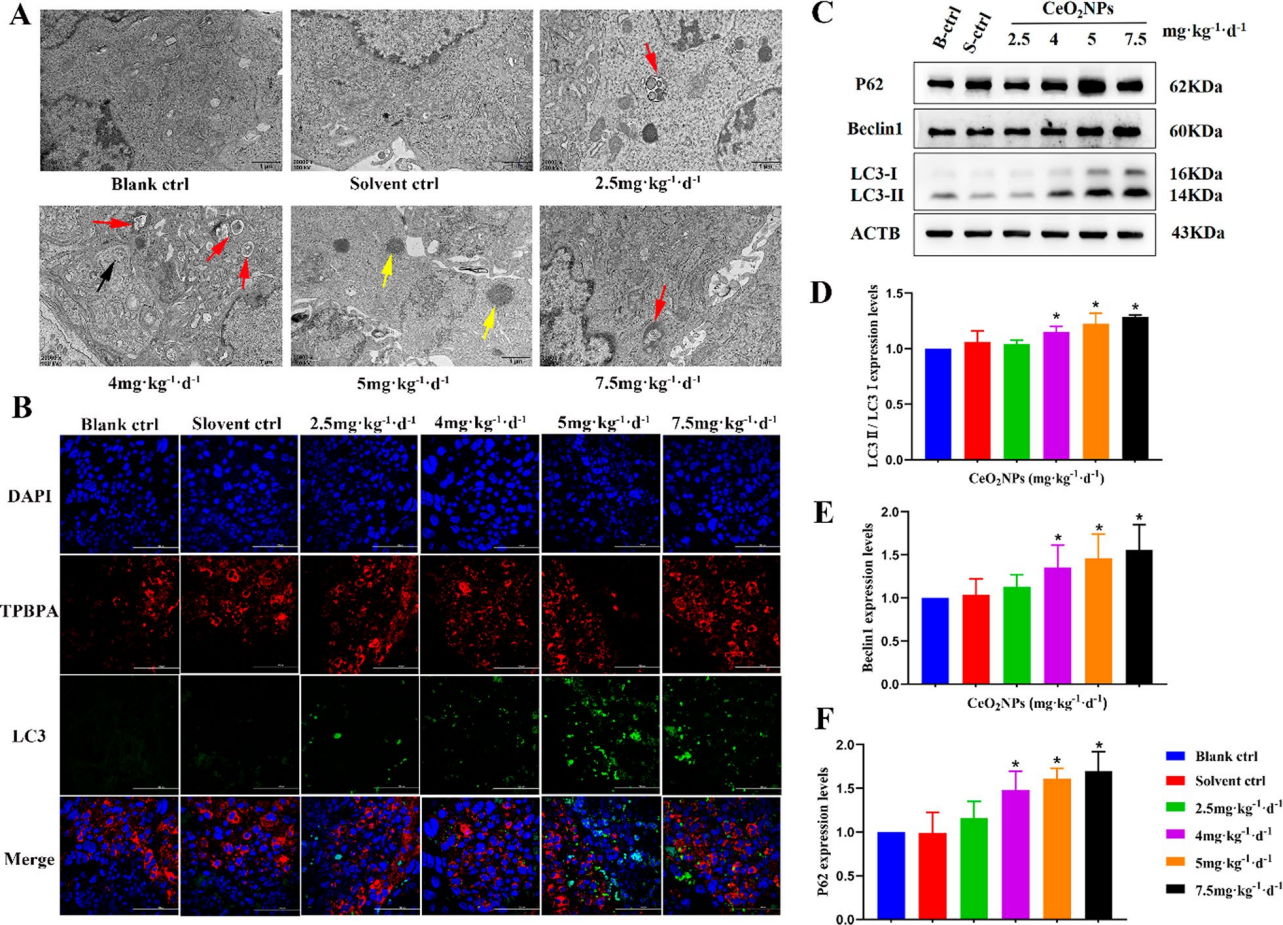
CeO_2_NPs exposure activated autophagy of placental trophoblast cells in mice. **A** Autophagy in placenta tissue on GD12 was observed by using TEM. Black arrow indicates autophagosome with a double membrane structure, red arrow indicates autolysosome with a single-layer membrane structure containing degraded organelles and yellow arrow indicates lysosome with a monolayer membrane structure (Scale bar = 1 μm). **B** Immunofluorescence was used to measure the expression of LC3 in placenta trophoblast cells exposed to different CeO_2_NPs groups on GD12. The red fluorescence represents spongiotrophoblast (Scale bar = 100 μm). The green fluorescence in merge image represents autophagy. **C** Western blot shows the expression of LC3II/I, Beclin1, and P62 in the blank control (B-ctrl), solvent control (S-ctrl) and CeO_2_NPs-treated groups on GD12. **D**–**F** Corresponding quantitative data of autophagy related proteins expression. *P < 0.05 compared with the control group

Autophagy dysfunction is an emerging mechanism of nanomaterials totoxicity. We investigated whether autophagy is involved in the effect of CeO_2_NPs exposure on placental trophoblast development. TEM results showed that the number of autophagosomes and lysosomes in CeO_2_NPs groups were significantly higher in the placental trophoblast than that in blank control and solvent control group (Fig. 3A). The immunofluorescence results of LC3, which indicated the accumulation of autophagosomes, were consistent with the TEM results and mainly expressed in the spongiotrophoblast layer (labeled with TPBPA) (Fig. 3B). Moreover, we examined the protein expression levels of LC3II/LC3I, Beclin1 and P62 (a substrate of autophagy) by Western Blot. Compared with that in the control group, increased LC3, Beclin1 and P62 levels were found in CeO_2_NPs-treated placental tissues in a dose dependent manner (Fig. 3C–F). These results elucidated that CeO_2_NPs exposure could activate autophagy in placental trophoblast. Therefore, we speculated that CeO_2_NPs-induced placental development abnormalities were probably caused by autophagy dysfunction.

### Effect of CeO_2_NPs on cell viability of HTR-8/SVneo

**Fig. 4:**
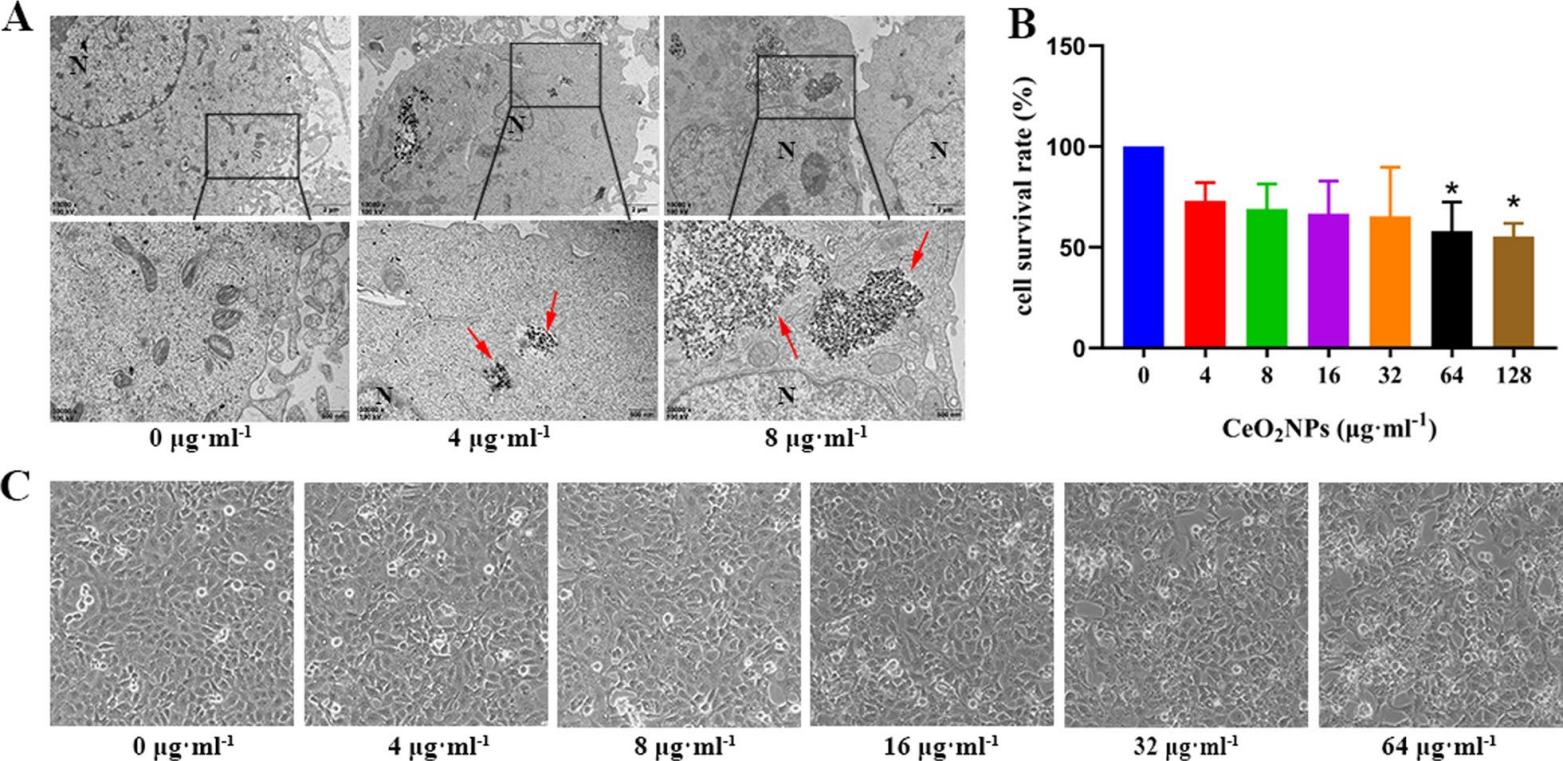
Cytotoxicity in HTR-8/SVneo cells induced by CeO_2_NPs. **A** Deposition of CeO_2_NPs in HTR-8/SVneo cells observed by TEM (Upper scale bar = 2 μm, lower scale bar = 500 nm). HTR-8/SVneo cells treated with 0, 4, and 8 μg ml^−1^ CeO_2_NPs for 24 h. CeO_2_NPs deposited in the cytoplasm (Red arrow). N: nucleus. **B** Cell viability was evaluated through the CCK-8 assay in HTR-8/SVneo cells upon exposure to CeO_2_NPs at different concentrations for 24 h. **C** Light microscope images with 200 magnification of CeO_2_NPs treated cells for 24 h. *P < 0.05 compared with the control group

HTR-8/SVneo cell, an immortalized first trimester trophoblast cell line, was used for further verification of the effect of CeO_2_NPs exposure on trophoblast and the related mechanisms in vitro. TEM showed that CeO_2_NPs were distributed in the cytoplasm of HTR-8/SVneo cells following treatment with CeO_2_NPs (Fig. 4A). The results of CCK-8 showed that the cell viability reduced significantly in the 64 μg ml^−1^ and 128 μg ml^−1^ CeO_2_NPs treatment groups (Fig. 4B). And the cell morphology did not change significantly after CeO_2_NPs exposure (Fig. 4C). The above results showed that CeO_2_NPs were able to be internalized by the cells, accumulated in the cytoplasm, and resulted in decreased cell viability without affecting cell morphology.

### CeO_2_NPs exposure inhibited cell migration and invasion of HTR-8/SVneo

**Fig. 5:**
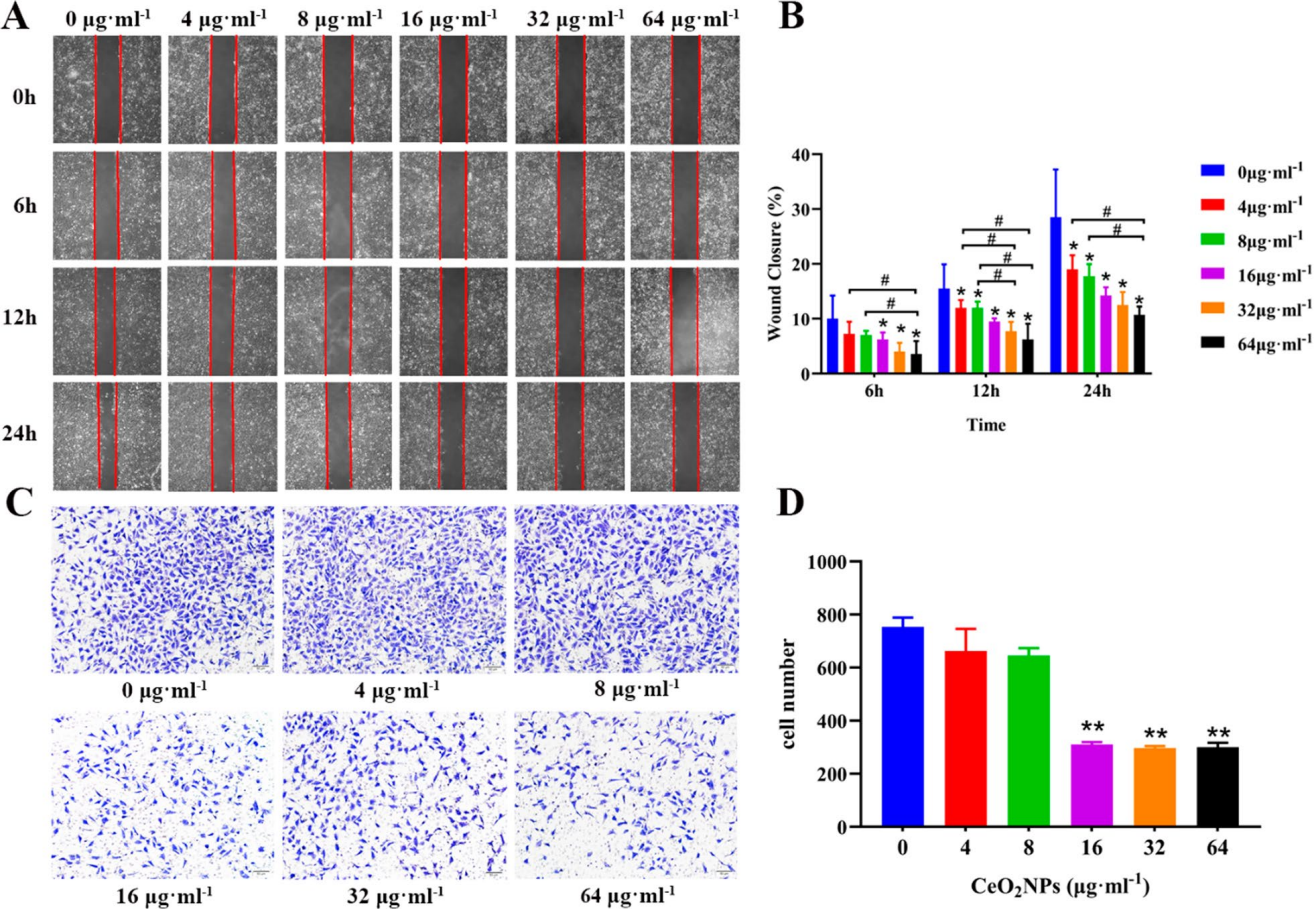
CeO_2_NPs exposure inhibited cell migration and invasion of HTR-8/SVneo cells. **A** Cell migration ability at 6 h, 12 h, and 24 h was assessed by cell scratch assay. **B** Quantification of the cell wound closure rate of 6 h, 12 h, and 24 h after CeO_2_NPs treatments as presented in **A**. **C** Cell invasion ability was estimated by transwell assay after different concentrations of CeO_2_NPs treatments and images were taken under light microscope (Scale bar = 50 μm). **D** Quantification of the invading cells, five insights were chosen in each group and values were presented as mean ± SE. *P < 0.05 compared with the control group. #P < 0.05 compared with the CeO_2_NPs-treated group, ** means P < 0.01

To assess the effects of CeO_2_NPs on trophoblast cell invasion and migration, cell scratch and transwell assay were respectively conducted. The effects of CeO_2_NPs on HTR-8/SVneo cells migration were detected by scratch experiments. The results revealed that 16, 32, and 64 μg ml^−1^ CeO_2_NPs treatments significantly attenuated the migratory ability of HTR-8/SVneo cells at 6 h compared with that in control group (Fig. 5A, B). As compared with that in control group, even 4 μg ml^−1^ CeO_2_NPs exposure group prominently decreased the wound closure rate of HTR-8/SVneo cells at 12 h and 24 h. 32 μg ml^−1^ CeO_2_NPs treatment remarkably reduced cell migration at 12 h, and 64 μg ml^−1^ CeO_2_NPs treatment significantly decreased cell migration at 12 h and 24 h compared with that in 4 and 8 μg ml^−1^ groups (Fig. 5B). Transwell invasion assay showed that 16, 32, and 64 μg ml^−1^ CeO_2_NPs treatment could decrease the number of HTR-8/SVneo invaded cells at 24 h compared with that of control (Fig. 5C, D). These results indicated that CeO_2_NPs exposure could impair the function of trophoblast.

### CeO_2_NPs exposure activated cell autophagy of HTR-8/SVneo by mTORC1 signaling pathway

**Fig. 6:**
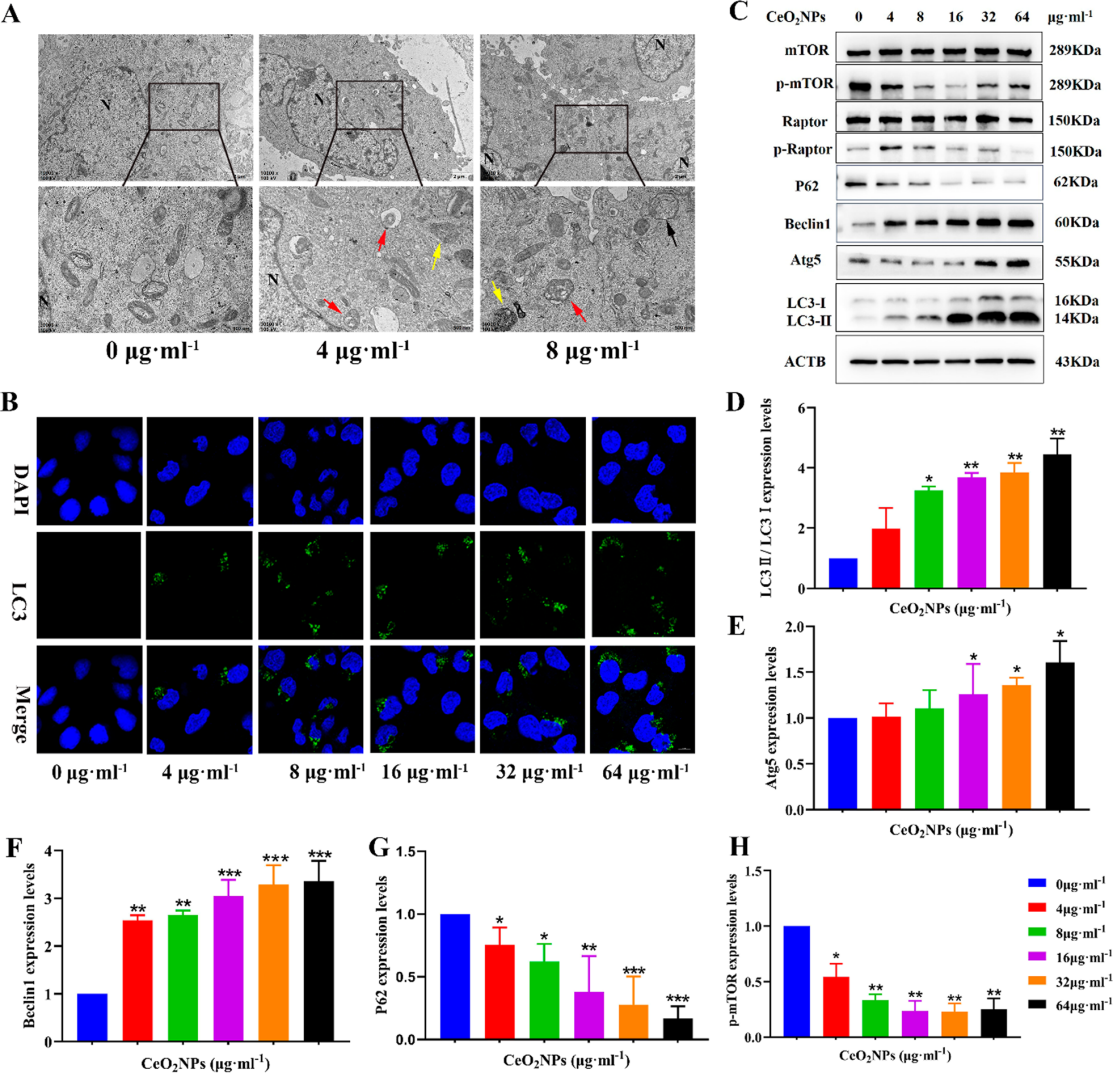
CeO_2_NPs exposure activated autophagy of HTR-8/SVneo cells by mTORC1 signaling pathway. **A** Autophagy was observed in HTR-8/SVneo cells treated with 4 and 8 μg ml^−1^ CeO_2_NPs for 24 h by using TEM. Black arrow indicates autophagosome with a double membrane structure, red arrow indicates autolysosome with a single-layer membrane structure containing degraded organelles and yellow arrow indicates lysosome with a monolayer membrane structure (N: nucleus, Upper scale bar = 2 μm, lower scale bar = 500 nm). **B** Immunofluorescence was used to measure the expression of LC3 in HTR-8/SVneo cells treated with different concentrations of CeO_2_NPs for 24 h (Scale bar = 10 μm). **C** Western blot showed the expression of LC3II/I, Beclin1, Atg5, P62, p-Raptor and p-mTOR in HTR-8/SVneo cells treated with CeO_2_NPs at different concentrations for 24 h. **D**–**H** Corresponding quantitative data of autophagy related proteins expression. *P < 0.05, **P < 0.01, ***P < 0.001, compared with the control group

As shown in Fig. 6A, the number of autophagosomes and autophagolysosomes were evidently increased following CeO_2_NPs treatment. Confocal microscopy showed that LC3 fluorescence level in HTR-8/SVneo cells increased significantly after 24 h treatment with CeO_2_NPs (Fig. 6B). Western Blot results showed that the protein levels of LC3, Beclin1 and Atg5 in the cells were significant increased after CeO_2_NPs treatment, while the expression level of P62 was decreased, indicating that CeO_2_NPs treatment promoted autophagy induction, but did not block autophagy flux (Fig. 6C–G). The accumulation of autophagosomes caused by CeO_2_NPs is due to the activation of autophagy rather than the blocking of autophagy flux. To further clarify the upstream regulator of CeO_2_NPs induced autophagy, we examined the mTOR signaling pathway by Western blot assay. The results revealed that the expression of mTOR and Raptor was down-regulated after CeO_2_NPs treatments (Fig. 6C, H). There was no difference in the expression level of proteins related to classical signaling pathways (PI3K/Akt/mTOR and AMPK/mTOR) among the groups, which suggested that the activated autophagy induced by mTOR inhibition depends on other regulatory pathways (Additional file 1: Figure S3A, B).

### Autophagy initiation inhibited by 3-MA partially reversed the cytotoxicity of CeO_2_NPs

**Fig. 7:**
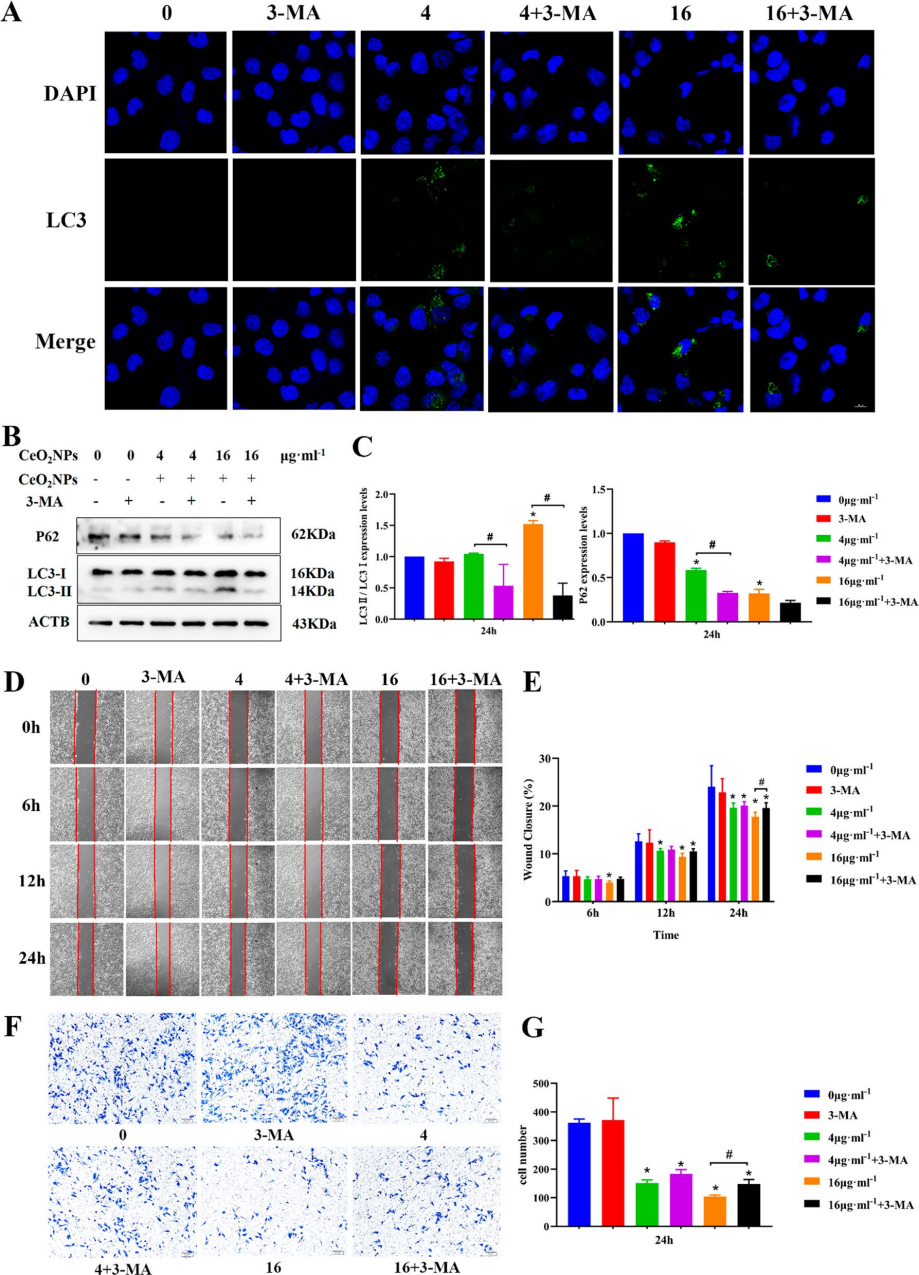
Autophagy initiation inhibited by 3-MA could partially reverse the cytotoxicity of CeO_2_NPs. HTR-8/SVneo cells were co-cultured with CeO_2_NPs at a final concentration of 0, 4, or 16 μg ml^−1^ for 24 h with or without 1 mM 3-MA. **A** Immunofluorescence showed the effect of 3-MA combined with CeO_2_NPs for 24 h on LC3 fluorescence level in HTR-8/SVneo cells (Scale bar = 10 μm). **B** The protein level of LC3-II/I and P62 were detected by Western Blot. **C** Corresponding quantitative data of autophagy related proteins expression. **D** Cell scratch assay showed the migration ability of HTR-8/SVneo cells at 6 h, 12 h, and 24 h after 24 h exposure of 1 mM 3-MA combined with CeO_2_NPs. **E** Quantification of the cell wound closure rate of 6 h, 12 h, and 24 h after 3-MA combined with CeO_2_NPs treatments as presented in **D**. **F** Cell invasion ability was estimated by transwell assay after 3-MA combined with different concentrations of CeO_2_NPs treatments and images were taken under light microscope (Scale bar = 50 μm). **G** Quantification of the invading cells number, five insights were chosen in each group and values were presented as mean ± SE, * and # both mean P < 0.05. *P < 0.05 compared with the control group. #P < 0.05 compared with the CeO_2_NPs-treated group

To further explore whether autophagy activation is indeed related to CeO_2_NPs-induced cytotoxicity, 3-MA (an autophagy inhibitor that can inhibit autophagosome formation) was employed. HTR-8/SVneo cells were treated with 0, 4, or 16 μg ml^−1^ CeO_2_NPs, in combination with or without 3-MA for 24 h. Fluorescence results showed that CeO_2_NPs combined with 3-MA treatment significantly reduced the LC3 green fluorescence levels in HTR-8/SVneo cells compared with that in CeO_2_NPs treatment groups (Fig. 7A). Cotreatment with 3-MA also resulted in a significant reduction of LC3 and P62 protein levels in HTR-8/SVneo cells (Fig. 7B, C). The scratch results showed that only 16 μg ml^−1^ CeO_2_NPs treatment significantly decreased the healing rate of HTR-8/SVneo cells at 6 h, while 4 μg ml^−1^ and 16 μg ml^−1^ CeO_2_NPs significantly decreased the healing rate of cells at 12 h and 24 h compared with that in blank control (Fig. 7D, E). 3-MA and CeO_2_NPs combined treatment markedly increased the 24 h wound healing rate of cells compared with that in CeO_2_NPs treatment group, while the migration rate was still lower than that of the blank control (Fig. 7E). Transwell assay indicated that combination of 3-MA with CeO_2_NPs evidently increased the number of cells crossing the chamber (Fig. 7F, G). These results showed that 3-MA treatment combined with CeO_2_NPs reversed the CeO_2_NPs-induced impairment of cell function. Autophagy activation may be the mechanism by which CeO_2_NPs exposure impairs trophoblast function.

### Blocking autophagy flow by CQ aggravated the cytotoxicity of CeO_2_NPs exposure

**Fig. 8:**
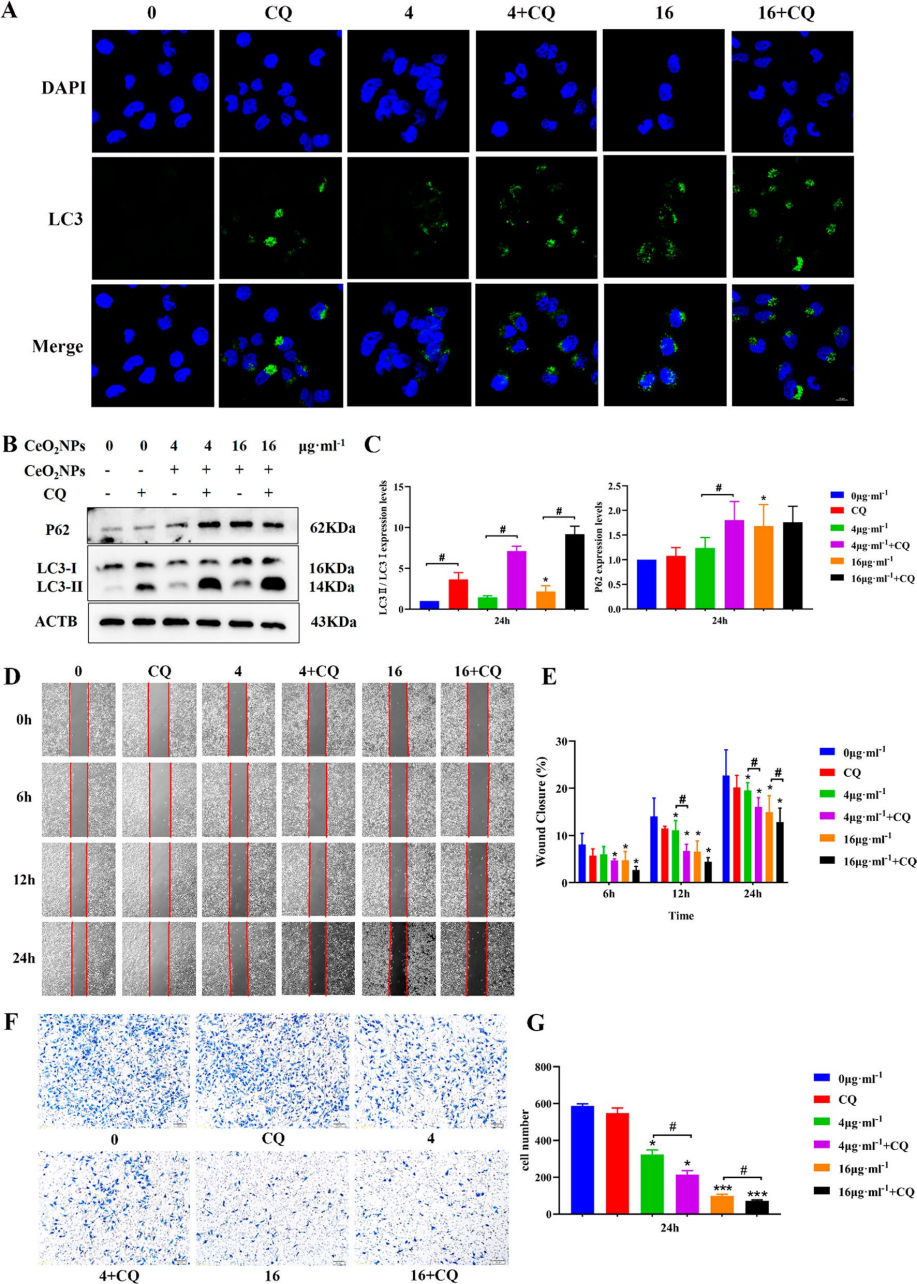
Blocking autophagy flow by CQ aggravated the cytotoxicity of CeO_2_NPs. HTR-8/SVneo cells were co-cultured with CeO_2_NPs at a final concentration of 0, 4, or 16 μg ml^−1^ for 24 h with or without 10 μM CQ. **A** Immunofluorescence showed the effect of CQ combined with CeO_2_NPs for 24 h on LC3 fluorescence level in HTR-8/SVneo cells (Scale bar = 10 μm). **B** The protein level of LC3-II/I and P62 were detected by Western Blot. **C** Corresponding quantitative data of autophagy related proteins expression. **D** Cell scratch assay shows the migration ability of HTR-8/SVneo cells at 6 h, 12 h, and 24 h after 24 h exposure of CQ combined with CeO_2_NPs. **E** Quantification of the cell wound closure rate of 6 h, 12 h, and 24 h after CQ combined with CeO_2_NPs treatments as presented in **D**. **F** Cell invasion ability was estimated by transwell assay after CQ combined with different concentrations of CeO_2_NPs treatments and images were taken under light microscope (Scale bar = 50 μm). **G** Quantification of the invading cells number, five insights were chosen in each group and values were presented as mean ± SE, * and # both mean P < 0.05. *P < 0.05 compared with the control group. *** means P < 0.001. #P < 0.05 compared with the CeO_2_NPs-treated group

Next, CQ (an autophagy inhibitor that can inhibit autophagosome–lysosome fusion and induce autophagosome accumulation) was employed to confirm the role of autophagy activation in CeO_2_NPs-induced cytotoxicity furtherly. HTR-8/SVneo cells were treated with CeO_2_NPs at 0, 4, or 16 μg ml^−1^, in the absence or presence of CQ, an inhibitor of autophagic flux or lysosomal degradation. Upon treatment with CeO_2_NPs and CQ, the LC3 green fluorescence evidently increased compared with CeO_2_NPs group (Fig. 8A). Western bolt results found that the levels of autophagy related proteins LC3 and P62 were significantly increased after 16 μg ml^−1^ CeO_2_NPs treatment compared with that in control group (Fig. 8B, C). Compared with a single treatment of CeO_2_NPs, co-treatment of CeO_2_NPs and CQ induced much higher LC3 and P62 protein levels in the HTR-8/SVneo cells (Fig. 8B, C). That indicated co-treatment with CeO_2_NPs and CQ could block autophagy flow and enhanced autophagy levels furtherly. The scratch results suggested that 4 μg ml^−1^ CeO_2_NPs significantly reduced the migration rate of cells at 12 h and 24 h, while 16 μg ml^−1^ CeO_2_NPs significantly decreased the migration rate of cells at 6 h, 12 h, and 24 h (Fig. 8D, E). In addition, we also found that the combination treatment of CQ with CeO_2_NPs significantly reduced the healing rate of cells at 12 h and 24 h compared with that in CeO_2_NPs group (Fig. 8E). The transwell assay results showed that the combination of CQ with CeO_2_NPs resulted in significantly fewer cells passing through the chamber compared with that in CeO_2_NPs treatment group, indicating that blocking autophagy flow by CQ furtherly weakened the invasion ability of HTR-8/SVneo cells (Fig. 8F, G). These results suggested that CQ combined with CeO_2_NPs further aggravated the functional damage of HTR-8/SVneo. These data validates excessive autophagy activation is the mechanism by which CeO_2_NPs exposure impairs trophoblast function, and autophagy may be a potential therapeutic target for the biological negative effect of CeO_2_NPs exposure.

### Mitochondrial autophagy of HTR-8/SVneo cells was not affected by CeO_2_NPs exposure

**Fig. 9:**
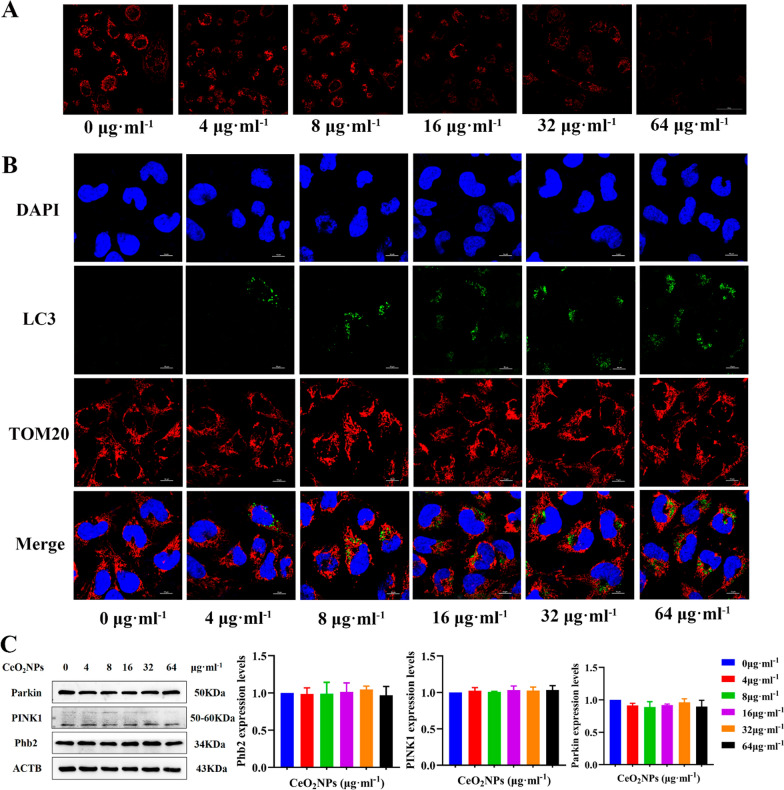
Mitochondrial autophagy of HTR-8/SVneo cells was not affected by CeO_2_NPs exposure. **A** MitoSOX fluorescence was used to measure the mitochondrial superoxide in HTR-8/SVneo cells treated with different concentrations of CeO_2_NPs for 24 h (Scale bar = 50 μm). **B** TOM20 and LC3 immunofluorescence staining of HTR-8/SVneo cells after exposure to different concentrations of CeO_2_NPs for 24 h (Scale bar = 10 μm). **C** Western blot showed the expression of Phb2, PINK1, and Parkin in HTR-8/SVneo cells treated with different concentrations of CeO_2_NPs for 24 h

The fluorescence intensity of MitoSOX (labeled mitochondria with high superoxide production) decreased after exposure to CeO_2_NPs (Fig. 9A), while the fluorescence signals of TOM20 (the marker of mitochondria) and LC3 did not overlap in HTR-8/SVneo cells (Fig. 9B). The protein levels of Phb2, Parkin, and PINK1 in cells did not change after CeO_2_NPs treatment (Fig. 9C). These results indicated that CeO_2_NPs exposure alleviated the higher mitochondrial pressure load of migrating cells which is independent of mitochondrial autophagy, suggesting that CeO_2_NPs exposure abated mitochondrial pressure load through other ways.

## Discussion

Nanomedicine offers unique advantages, such as using liposomes as nanocarriers targeting specific cells or tissues, which can limit and target drug delivery, even without fetal contact [35, 36]. With the advance of nanotechnology, CeO_2_NPs have been widely utilized as engineered nanomaterials and attracted great interests in a variety of biomedical applications, especially in bioimaging, drug delivery and cancer therapy [37–39]. But there are few studies on the reproductive effects of CeO_2_NPs, which limits its further development in biomedical application. A few studies have found that placental translocation occurred during pregnancy with the administration of inorganic nanoparticles such as gold and carbon nanotubes [36, 40, 41]. The ability of nanoparticles to cross the placenta is size-dependent [42]. Similarly, our TEM results showed that CeO_2_NPs accumulation occurred in the cytoplasm of placental trophoblast cell on GD12 after CeO_2_NPs exposure, indicating that CeO_2_NPs (3–5 nm) could cross placental barrier.

Placenta, the earliest organ formed in the embryonic development of mammals, is a transient organ at the maternal–fetal interface during gestational that facilitates the substance exchange between fetus and mother as well as fetal waste metabolic, and is essential for maintaining normal pregnancy and fetal growth as a protective barrier. Survival and growth of the fetus are critically dependent on the placenta. In mice, the formation of placental trophoblast begins on GD5 with the implantation of blastocyst. The trophectoderm layer separate from the inner cell mass by this time. On GD10, the chorioallantoic attachment occurs, and the labyrinth trophoblast begins to form, structurally supported by the spongiotrophoblast from the ectoplacental cone, in which all the exchange of gases and nutrients takes place. On GD12, the structure of mouse placenta is basically formed. The mature murine placenta is comprised of four layers: maternal decidua, trophoblast giant cell, spongiotrophoblast and labyrinth. In this study, we found that in 5 and 7.5 mg kg^−1^ day^−1^ CeO_2_NPs treatment groups, the area of labyrinth layer decreased, which had an adverse effect on placental development, may result from the abnormal development of extraembryonic ectoderm on GD9, and inhibition of ectoplacental cone invasion into maternal decidua and chorioallantoic attachment on GD10 after exposure to CeO_2_NPs.

Autophagy activation in placental trophoblast after CeO_2_NPs exposure was observed, thus human chorionic trophoblast HTR-8/SVneo was employed to clarify the mechanism in vitro. CeO_2_NPs were able to be internalized by the cells, accumulated in the cytoplasm, and resulted in decreased cell viability without affecting cell morphology. Autophagy is a dynamic process of lysosome-mediated degradation of cellular components or foreign bodies, including the initiation, formation, maturation and degradation of autophagosomes, which is also called autophagic flux. Thus, autophagy induced by CeO_2_NPs may be an attempt to degrade what is perceived by the cells as foreign or aberrant. Autophagy usually acts as a protective process, which sequestered and degraded damaged organelles or unnecessary proteins, to maintain cellular homeostasis [43]. Nevertheless, excessive autophagy activation and blockade of autophagic flux cause cell dysfunction. At present, LC3 lipidation has been commonly employed to measure autophagy activity. However, an increase in LC3-II may result from either an enhancement of autophagosomal formation or inhibition of autophagosomal degradation, or may be caused by autophagy-independent mechanisms [44]. Therefore, both generation and degradation of autophagosomes must be taken into consideration when LC3-II is used to evaluate autophagy activity. The accumulation of autophagosomes caused by CeO_2_NPs is due to the activation of autophagy rather than the blocking of autophagy flux, and is represented by increased LC3 protein levels and decreased P62 protein levels. Furthermore, our results showed that 3-MA treatment combined with CeO_2_NPs reversed the CeO_2_NPs-induced impairment of cell function, while CQ combined with CeO_2_NPs further aggravated the functional damage of HTR-8/SVneo. The data indicates that excessive autophagy activation is the mechanism by which CeO_2_NPs exposure impair trophoblast function, and autophagy may be a potential therapeutic target for the biological negative effect of CeO_2_NPs exposure.

Autophagy is regulated by multiple signaling pathways, such as nutrient Signaling, TOR complex1, and so on [45]. Among the signaling pathways implicated in the control of autophagy, the best characterized regulator for autophagy is mammalian target of rapamycin complex1 (mTORC1) that consists of mTOR, Raptor (regulatory associated protein of mTOR) and MLST8, and is the central regulator of many metabolic pathways to regulate autophagy, cell growth, cell proliferation as well as other cellular activities [46]. It is well-known that mTORC1 is in active state and inhibits autophagy at physiological conditions, while autophagy is activated by inhibiting mTOR during starvation or stress conditions [46]. In our study, CeO_2_NPs inhibit the phosphorylation of mTOR in HTR-8/SVneo cells, indicating that CeO_2_NPs suppress mTOR activity. It is likely that CeO_2_NPs activate autophagy by inhibiting the mTOR pathway. PI3K/Akt/mTOR and AMPK/mTOR signaling have been investigated in numerous studies as classical autophagy signaling pathways. Interestingly, mTOR inhibition caused by CeO_2_NPs exposure on HTR-8/SVneo cells was not regulated by these two classical signal pathways. The upstream regulation pathway of mTOR in HTR-8/SVneo cells needs to be further studied.

Compared with non-migrating cells, migrating cells need to consume more energy to support migration. Thus, migrating cells have higher respiratory rate, more ROS production and higher mitochondrial pressure load. Migrating cells selectively remove damaged mitochondria through a variety of ways to maintain mitochondrial homeostasis [47]. We found that the number of damaged mitochondria in HTR-8/SVneo cells decreased after CeO_2_NPs treatment. Selective autophagy of mitochondria, known as mitophagy, is also an important mitochondrial quality control mechanism that eliminates damaged mitochondria by the autophagy machinery. We found that CeO_2_NPs exposure alleviated the higher mitochondrial pressure load of HTR-8/SVneo without mitophagy activation. It is suggested that CeO2NPs exposure alleviated mitochondrial pressure load through other ways. It has been reported that upon exposure to mild mitochondrial stresses, the damaged mitochondrion is transported into migrasomes and subsquently disposed of from migrating cells, a process called mitocytosis, in which migrating cells selectively remove damaged mitochondria to maintain homeostasis [47]. The role of mitocytosis, a migrasome-mediated mitochondrial quality-control process, in HTR-8/SVneo cells exposed to CeO_2_NPs is worth further exploring.

## Conclusion

In summary, we found that CeO_2_NPs with diameter of 3–5 nm could cross the placental barrier and deposit in the cytoplasm of trophoblast cell, causing abnormal placental development. The exposure of CeO_2_NPs during pregnancy led to autophagy activation in placental trophoblast cells, indicating that autophagy dysfunction may be the cause of abnormal placental development. On this basis, the safe application range of CeO_2_NPs (less than 4 mg kg^−1^ day^−1^) in mice during pregnancy was screened, which provided experimental basis for evaluating the risk of pregnant female exposed to CeO_2_NPs. Exposure to CeO_2_NPs inhibited the migration and invasion of HTR-8/SVneo and activated autophagy excessively through mTORC1 signaling pathway. The mechanism of CeO_2_NPs exposure affecting trophoblast cell function was firstly clarified from the perspective of autophagy, providing clues for the biological negative effect prevention target of pregnancy exposure. Our study shed new light on the mechanism underlying the toxicity induced by rare-earth nanoparticle in the reproductive system.

## Materials and methods

### The preparation and characterization of CeO_2_NPs

The preparation of CeO_2_NPs was performed as previously described [48]. Briefly, the microemulsion method was used to prepare CeO_2_NPs. Non-agglomerated cerium oxide nanoparticles were dissolved in the upper toluene solution. The pure CeO_2_NPs was extracted from toluene by washing with ammonia water and anhydrous ethanol for 1 time, anhydrous ethanol for 3 times, and then ultra-pure water for 1 time. The nano cerium dioxide was then prepared into 1 mg ml^−1^ solution with ultrapure water for animal experiments. The field emission transmission electron microscope (FEI Tecnai G2 F20) was used for determining the size and morphology of the CeO_2_NPs.

Female and male BALB/c mice (8–10 week-old) used in this study were purchased from Beijing Vital River Laboratory Animal Technology Co., Ltd (Beijing, China). The mice were housed in the Animal Facility of Chongqing Medical University under standard conditions of constant temperature (22 ± 2 °C), humidity (50%), and a 12-h light/dark cycle with enough normal chow diet and water provided ad libitum (five females per cage). All animal procedures were approved by the Chongqing Medical University Animal Care and Use Committee and followed the principles in the Guide for the Care and Use of Laboratory Animals. After a 2-week adaptation period, female mice were mated overnight with male (two females and one male per cage), and the appearance of vaginal plug was considered to be gestational day 1 (GD1). According to the recommended dose for tumor treatment [49], pregnant mice were randomly divided into blank control, solvent control (ultra-pure water) and CeO_2_NPs-exposed groups (2.5, 4, 5, 7.5, and 10 mg kg^−1^ day^−1^, at least seven mice in each group). The mice were injected intravenously with different doses of CeO_2_NPs once a day at 8 am on GD5, GD6, and GD7 through tail vein. Then, the mice were anesthetized and sacrificed to collect tissues on GD8, GD9, GD10, and GD12. Uterine appearance images were collected and uterine wet weight was measured on GD8, GD9, and GD10. Placenta and embryos images or weights were collected on GD12 for analysis the pregnancy status. These tissues were partially fixed in 4% paraformaldehyde solution or flash-frozen in liquid nitrogen until use.

### Transmission electron microscopy

Fresh placental tissues were immediately cut into tissue blocks of 1mm^3^ and stored in glutaraldehyde fixation solution at 4 °C within 1 min. The HTR-8/SVneo cells were treated with CeO_2_NPs(0, 4, and 8 μg ml^−1^) for 24 h and washed 3 times by phosphate-buffered saline (Boster, China). Then, CeO_2_NPs-treated cells were collected into a 2 ml microcentrifuge tube following pancreatin digestion and fixed in glutaraldehyde. Transmission electron microscopy (TEM) was performed by the College of Life Sciences of Chongqing Medical University.

### Hematoxylin and eosin staining

The uterus and placenta tissues fixed in 4% paraformaldehyde solution were dehydrated and paraffin embedded. Paraffin sections (4 μm) were deparaffinized, hydrated and stained with HE (Jiancheng, China). And the images were then photographed by using an Olympus B × 50 (Olympus) photo microscope. The areas of maternal decidual, spongiotrophoblast layer and labyrinth layer in the placenta on GD12 were quantitatively analysed with Image J (National Institutes of Health).

### Cell culture and treatment

The HTR-8/SVneo extravillous trophoblast cell line (CTCC-400–0143, Meisen CTCC, China) was cultured in 10% RPMI-1640 medium (Sigma, USA) at 37 °C, 5%CO_2_. CeO_2_NPs was dissolved in RPMI-1640 medium at a concentration of 1 mg ml^−1^. HTR-8/SVneo cells were respectively treated with 4, 8, 16, 32, or 64 μg ml^−1^ CeO_2_NPs for 24 h, while blank control group without any treatment [50]. Referring to the existing reports, 1 mM 3-MA (M9281, Sigma, USA) or 10 μM CQ (CAS:54-05-7, MCE, China) was added to the HTR-8/SVneo cells in combination with CeO_2_NPs or not for 24 h [32, 51].

### Cell viability assay

HTR-8/SVneo cells were seeded in 96-well plates at a density of 5000 cells/well, being cultured for 24 h before adding different concentrations of CeO_2_NPs (0, 4, 8, 16, 32, 64,128, or 256 μg ml^−1^). Following another 24 h incubation, 10 µl Cell Counting Kit-8 (CCK-8, Dojindo, Japan) solution was added and cells were incubated at 37 °C for 1–4 h. Then the absorbance at 450 nm was measured with a microplate reader. The level of cell viability was calculated according to the manufacturer's instructions.

### Immunofluorescence analysis

Placenta paraffin sections underwent dewaxing, hydration and antigen retrieval. The sections were blocked by ADB and incubated in primary antibody (LC3: Cell Signal Technology, USA, #83,506, TPBPA: Abcam, ab104401, MCT1: Signalway Antibody, #38,537, MCT4: Santa, sc-376140) followed by secondary antibody incubation. Cultured HTR-8/SVneo cells were treated with CeO_2_NPs (0, 4, 8, 16, 32, and 64 μg ml^−1^) for 24 h and then were fixed with 4% paraformaldehyde for 10 min. After treatment with 0.5% Triton X-100, the cells were treated with primary antibody (LC3: Cell Signal Technology, USA, #83,506, TOM20: Proteintech, 11,802–1-AP) diluted by PBS and incubated at 4 °C overnight. Then the cells were incubated with fluorescein isothiocyanate-labelled mouse IgG (Zhongshan, China) or Cy3-labeled goat antibody IgG (Beyotime, China) for 1 h and DAPI (Beyotime, China) for 10 min. Finally, confocal fluorescence microscopy (BX43, Olympus) was performed to observe the slides after sealed with fluorescent anti-quenching reagents (Beyotime, China).

### Migration capability detection by Scratch assay

Cell migration capacity was determined by a scratch-wound healing assay. HTR-8/SVneo cells were cultured on six-well plates for 24 h and treated with different concentrations of CeO_2_NPs for another 24 h when the cell density was about 75%. When the cell grew to 100% confluency, a sterile 1 ml pipette tip was used to perform a scratch. Serum-free RPMI-1640 medium were used to culture HTR-8/SVneo cells for 24 h. The images of scratched areas were taken by microscope (Nikon, Japan) at 0 h, 6 h, 12 h, and 24 h. And the wound healing rates of HTR-8/SVneo cells at 6 h, 12 h, and 24 h was measured using Image J. For instance, the wound closure rates of 6 h is equal to the difference of scratch area between 0 and 6 h**/**scratch area of 0 h*100%. Cell mobility was determined with the following formula:

$$ {\text{Mobility }}\left( \% \right) \, = \, \left( {{\text{scratch area at T}}_{0} - {\text{ scratch area at T}}} \right)/{\text{scratch area at T}}_{0} \times { 1}00\% . $$

### Invasion capacity detection by Transwell assay

After treatment with different concentrations of CeO_2_NPs for 24 h, HTR-8/SVneo cells (5*10^4^, counted by cell counter, Nikon, Japan) were seeded into the transwell chamber for 24 h with 200 µl serum-free medium in the upper chamber pre-coated with Matrigel (BD Matrigel Matrix Cat. No. 356234) and 500 µl containing 20% fetal bovine serum (PAN, Germany) medium in the lower chamber. The cells were fixed with iced methanol for 15 min at 4 °C, stained with crystal violet (Beyotime, China) for 30 min and counted by Image J to assess cell invasive capability.

### Western blot analysis

Total proteins in placenta tissue or cells were extracted with RIPPA lysis buffer (Beyotime, China) and quantified the concentration by BCA Protein Assay Kit (Beyotime, China). Protein samples were separated using 10% or 12% SDS-PAGE and transferred onto PVDF membranes (Bio-Rad, Canada). Membranes were blocked with 5% milk (Boster, China), incubated overnight at 4 °C with primary antibody and further incubated with HRP-labelled corresponding source of secondary antibody (Boster, China) at 37 °C for 1 h. Primary antibodies (Zhongshan, China: ACTB, CST: LC3, #83,506, Atg5, #12,994, Beclin-1, #3495, P62, #5114, mTOR, #2983, p-mTOR, #5536, Akt, #4691, p-Akt, #4060, Raptor, #2280, p-Raptor, #2083, AMPK, #5832, p-AMPK, #2535, Santa: PINK1, sc-517353, Parkin, sc-32282, Phb2, sc-133094) were diluted in 5% milk in PBST. The band intensity was visualized using enhanced chemiluminescence reagent (Bio-Rad, USA) and quantified by Image J software.

### Statistical analysis

Statistical analysis was performed with SPSS 22.0. Quantitative data were presented as means ± standard deviation (SD), and all experiments were repeated independently at least three times. One Way ANOVA with Bonferroni post hoc was used to compare all treatment groups to control group. The elements of graphical abstract are derived from BioRender.com. And all datas were plotted using GraphPad Prism version 8.0. P < 0.05 was considered as statistically significant.

## Supplementary Information

**Additional file 1: Figure S1.** Effects of CeO_2_NPs exposure on pregnancy status in mice. (A) The maternal body weight, (B) the uterine weight and (C) the ratio of uterine**/**maternal weight on GD8, GD9, GD10, and GD12. (D)The uterine appearance on GD8 and GD9 (n = 7, Scale bar = 1 cm). **Figure S2.** Effects of CeO_2_NPs exposure on early placental development in pregnant mice. (A) HE staining of uterine tissue on GD8 observed under light microscope (Scale bar = 200 µm). (B) HE staining of uterine tissue on GD9 observed under light microscope (Upper scale bar = 200 μm, lower scale bar = 50 µm) a: ectoplacental cone (EPC), b: extraembryonic ectoderm. (C) The area of ectoplacental cone and extraembryonic ectoderm. Values are expressed as the mean ± SD. *p < 0.05 compared with the control group. **Figure S3.** CeO_2_NPs exposure activated autophagy of HTR-8/SVneo cells by mTORC1 signaling pathway. (A) Western blot showed the expression of AKT, p-PI3K 85, and p-PI3K 110 in HTR-8/SVneo cells treated with CeO_2_NPs at different concentrations for 24 h. (B) Western blot showed the expression of AMPK and p-AMPK in HTR-8/SVneo cells treated with CeO_2_NPs at different concentrations for 24 h.

## Data Availability

All the original data are available upon reasonable request for correspondence authors.

## Abbreviations

CeO_2_NPs: Cerium dioxide nanoparticles
PM2.5: Fine particulate matter
HTR-8/SVneo: Human chorionic trophoblast cell line
mTORC1: Mammalian target of rapamycin complex1
FE-TEM: Field emission transmission electron microscopy
TEM: Transmission electron microscopy
N: Nucleus
GD: Gestational day
HE staining: Hematoxylin and eosin (H&E) staining
EPC: Ectoplacental cone
Ch: Chorionic ectoderm
MCT1: Monocarboxylate transporters 1
MCT4: Monocarboxylate transporters 4
SynT-1: Syncytiotrophoblast-1
SynT-2: Syncytiotrophoblast-2
MD: Maternal decidual
SP: Spongiotrophoblast
Lab: Labyrinth
TPBPA: Trophoblast specific protein alpha
LC3: Microtubule-associated protein 1 light chain 3 alpha
Atg5: Autophagy-related gene
P62: Sequestosome 1
B-ctrl: Blank control
S-ctrl: Solvent control
CCK-8: Cell Counting Kit-8
PBS: Phosphate buffered saline
PBST: Tris-buffered-saline with tween
PVDF: Polyvinylidene fluoride
3-MA: 3-Methyladenine
CQ: Chloroquine
SD: Standard deviation
mM: Millimolar
μM: Micromolar
μl: Microliter
h: Hour
KDa: Kilodalton
