# Supplementary material for: Maternal exposure to CeO2NPs derails placental development through trophoblast dysfunction mediated by excessive autophagy activation
Source: J Nanobiotechnology. 2022 Mar 15;20:131. doi: 10.1186/s12951-022-01334-8 (PMC8922923; doi:10.1186/s12951-022-01334-8)

Additional Information

Maternal exposure to CeO_2_NPs derails placental development through trophoblast dysfunction mediated by excessive autophagy activation

**Figure S1.** Effects of CeO_2_NPs exposure on pregnancy status in mice. (A) The maternal body weight, (B) the uterine weight and (C) the ratio of uterine/maternal weight on GD8, GD9, GD10, and GD12. (D)The uterine appearance on GD8 and GD9 (n=7, Scale bar=1 cm).

**
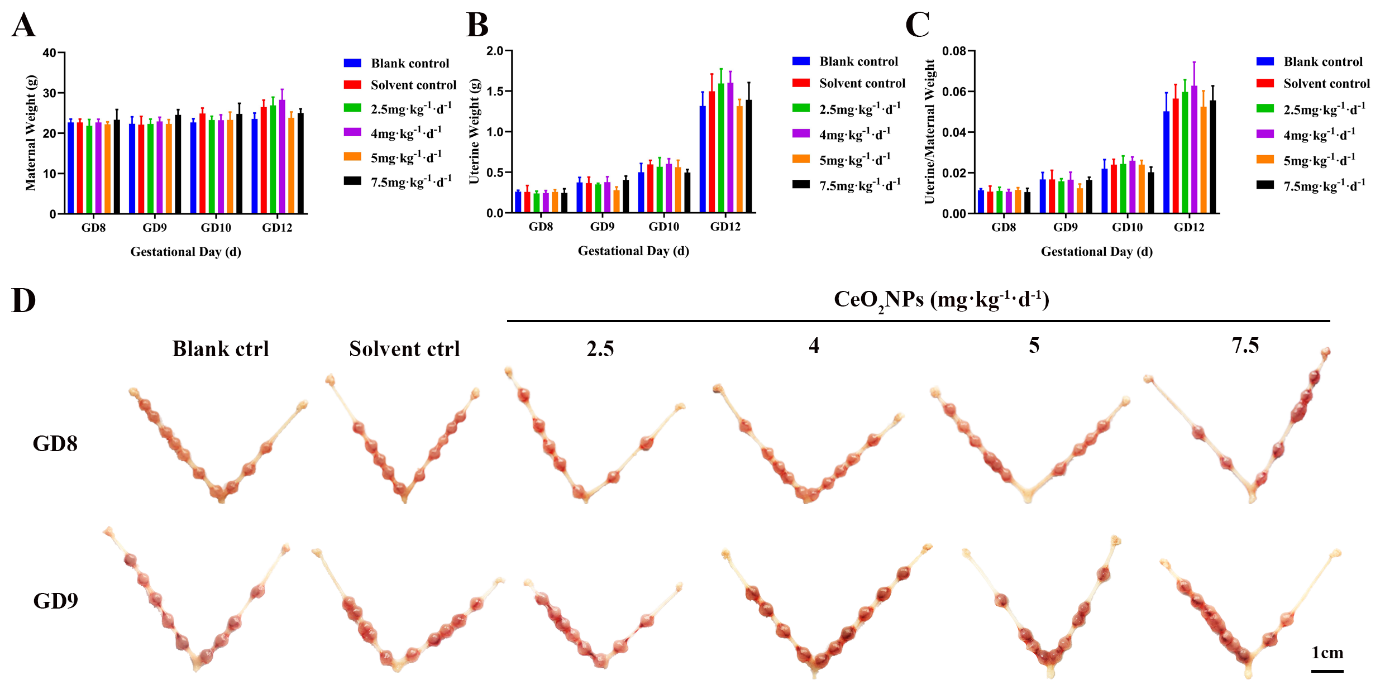
**

**Figure S2.** Effects of CeO_2_NPs exposure on early placental development in pregnant mice. (A) HE staining of uterine tissue on GD8 observed under light microscope (Scale bar=200 µm). (B) HE staining of uterine tissue on GD9 observed under light microscope (Upper scale bar=200μm, lower scale bar=50µm) a: ectoplacental cone (EPC), b: extraembryonic ectoderm. (C) The area of ectoplacental cone and extraembryonic ectoderm. Values are expressed as the mean ± SD. *p < 0.05 compared with the control group.


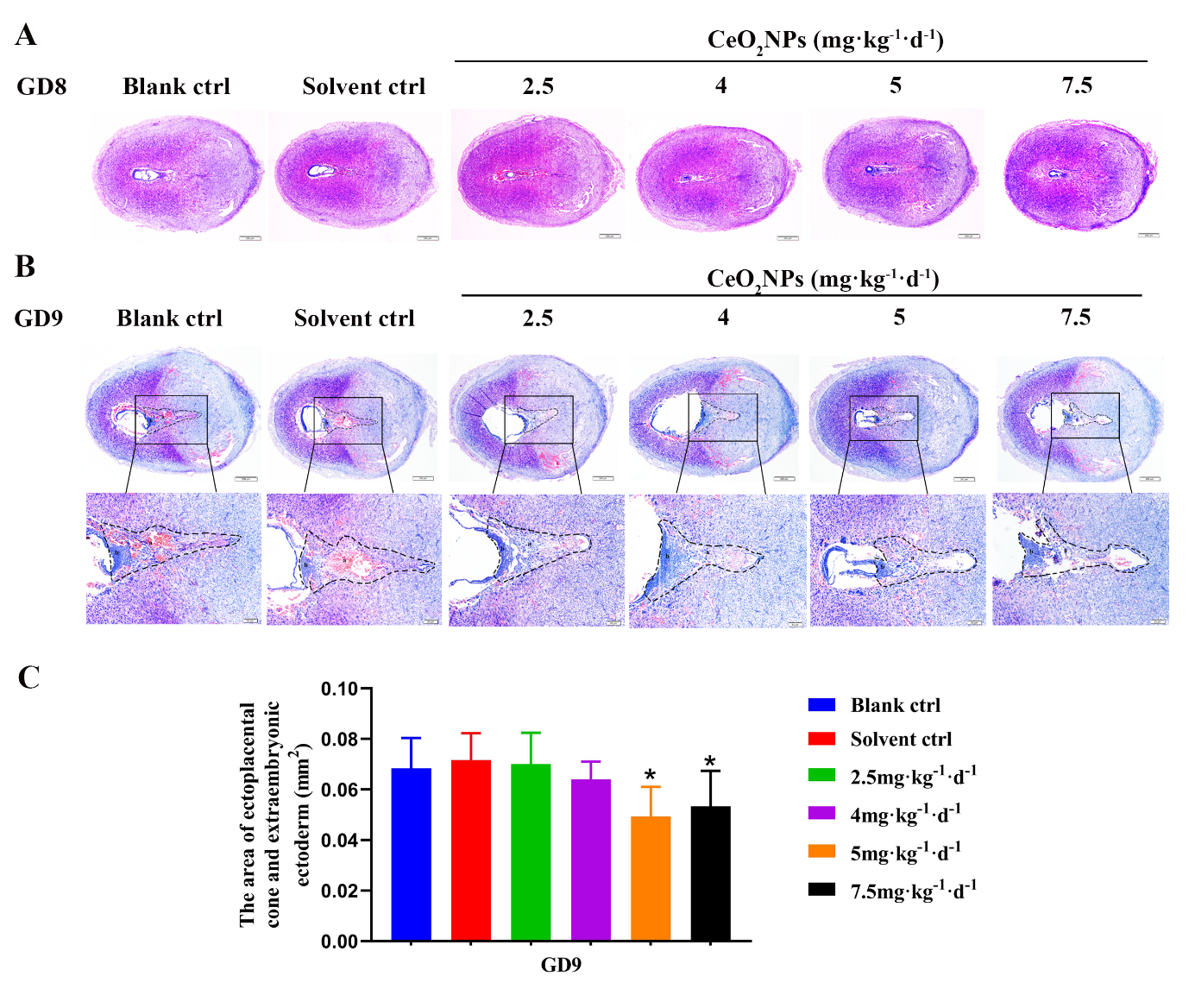


**Figure S3.** CeO_2_NPs exposure activated autophagy of HTR-8/SVneo cells by mTORC1 signaling pathway. (A) Western blot showed the expression of AKT, p-PI3K 85, and p-PI3K 110 in HTR-8/SVneo cells treated with CeO_2_NPs at different concentrations for 24h. (B) Western blot showed the expression of AMPK and p-AMPK in HTR-8/SVneo cells treated with CeO_2_NPs at different concentrations for 24h.


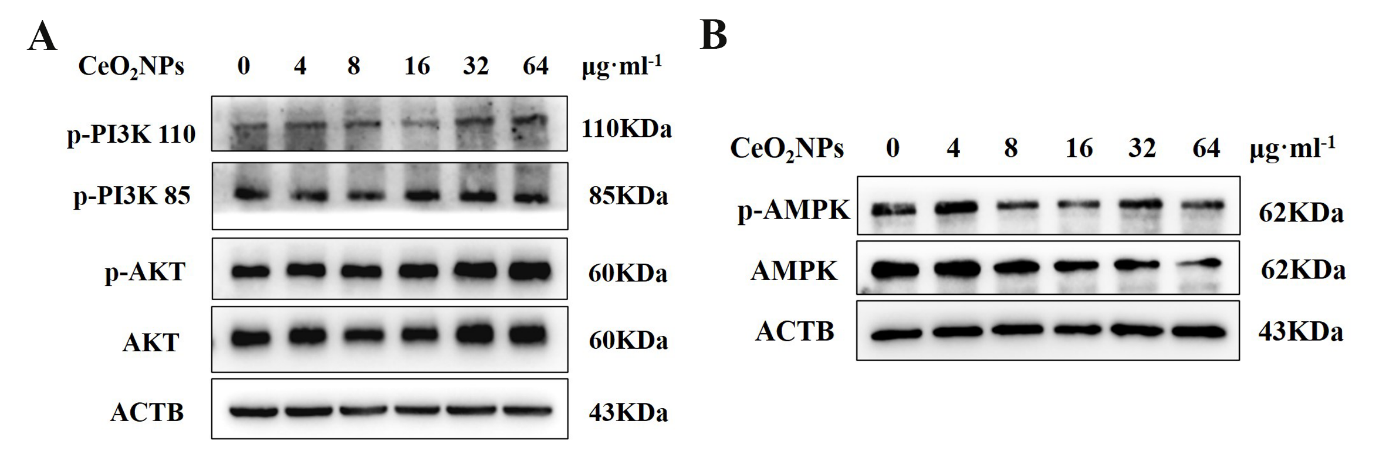

Supplement: Supplementary file 1 — Additional file 1: Figure S1. Effects of CeO2NPs exposure on pregnancy status in mice. (A) The maternal body weight, (B) the uterine weight and (C) the ratio of uterine/maternal weight on GD8, GD9, GD10, and GD12. (D)The uterine appearance on GD8 and GD9 (n = 7, Scale bar = 1 cm). Figure S2. Effects of CeO2NPs exposure on early placental development in pregnant mice. (A) HE staining of uterine tissue on GD8 observed under light microscope (Scale bar = 200 µm). (B) HE staining of uterine tissue on GD9 observed under light microscope (Upper scale bar = 200 μm, lower scale bar = 50 µm) a: ectoplacental cone (EPC), b: extraembryonic ectoderm. (C) The area of ectoplacental cone and extraembryonic ectoderm. Values are expressed as the mean ± SD. *p < 0.05 compared with the control group. Figure S3. CeO2NPs exposure activated autophagy of HTR-8/SVneo cells by mTORC1 signaling pathway. (A) Western blot showed the expression of AKT, p-PI3K 85, and p-PI3K 110 in HTR-8/SVneo cells treated with CeO2NPs at different concentrations for 24 h. (B) Western blot showed the expression of AMPK and p-AMPK in HTR-8/SVneo cells treated with CeO2NPs at different concentrations for 24 h. [file 12951_2022_1334_MOESM1_ESM.docx]
